# First Dominique Dormont international conference on "Host-pathogen interactions in chronic infections – viral and host determinants of HCV, HCMV, and HIV infections"

**DOI:** 10.1186/1742-4690-2-24

**Published:** 2005-04-06

**Authors:** Elisabeth Menu, Mickaela C Müller-Trutwin, Gianfranco Pancino, Asier Saez-Cirion, Christine Bain, Geneviève Inchauspé, Gabriel S Gras, Aloïse M Mabondzo, Assia Samri, Françoise Boutboul, Roger Le Grand

**Affiliations:** 1 Laboratoire de Biologie des Rétrovirus, Institut Pasteur, 25–28 rue du Dr Roux, 75015 Paris, France; 2 FRE 2736, CNRS-BioMérieu, Immunothérapie des maladies Infectieuses Chroniques, Ecole Normale Supérieure, 46 Allée d'Italie 69 364 Lyon Cédex 07, Fance; 3 CEA, Service de Neurovirologie, UMRE1 Université Paris XI, 18 route du Panorama, 92265 Fontenay-aux-Roses, Cedex, France; 4 CEA, Service de Pharmacologie et d'Immunologie, 91191 Gif sur Yvette cedex, France; 5 Laboratoire d'Immunologie Cellulaire et Tissulaire, INSERM U543 – Université Paris VI Pierre et Marie Curie Hôpital Pitié-Salpêtrière, 83 Bld de l'Hôpital, 75651 PARIS Cédex 13, France

## Abstract

The first Dominique Dormont International Conference on "Viral and host determinantsof HCV, HCMV, and HIV infections "was held in Paris, Val-de-Grâce, on December 3–4, 2004. The following is a summary of the scientific sessions of this meeting ().

## Background

The Dominique Dormont Conferences provide an international forum for the promotion of exchanges between clinicians and fundamental scientists, including team leaders and young researchers, involved in interdisciplinary research on chronic infections. They provide an occasion for researchers with common interests to get together for two or three days of synthesis and intense discussion on the most recent advances in their field, to crystallize new research directions and collaborations. Contacts with young scientists are strongly encouraged during the conference and such exchanges are facilitated by limiting attendance at each conference to 150 participants, on a first-come, first-served basis. The participants include prestigious invited speakers for state-of-the-art introductions to each scientific session, followed by abstract-driven talks, most presented by young investigators. Abstract-driven poster sessions also provide a space for scientific exchanges between team leaders and young researchers. An International Scientific Program Committee establishes the final program and abstracts are selected according to the highest scientific standards.

The Dominique Dormont International Conferences will be held every year on different specific thematic topics related to "Host-Pathogen Interactions in Chronic Infections". In December 2004, the topic of the first conference was "Viral and Host Determinants of HCV, HCMV and HIV Infections", considering the pathogenesis of these chronic viral infections as a priority topic for the development of interdisciplinary research and collaborations between clinicians and fundamental scientists worldwide. The International Scientific Program Committee selected original presentations on six topics of particular interest in the field. Over the course of two days, stimulating exchanges and discussions between team leaders and young researchers, highlighting key issues in this research field, were achieved, raising hopes of opening the way to further international investigations on interactions between host and viral determinants.

## Summary of the scientific sessions

### Session 1: Receptor and viral entry

***Chairs: ***F. Arenzana and U. Koszinowski; ***Keynote Lecture: ***"New aspects on hepatitis C virus replication, assembly and entry into host cells" by R. Bartenschlager.

#### Factors enhancing viral entry

Viral entry depends in part on expression of the corresponding cellular receptors. The CD4 molecule has been identified as the receptor for HIV, whereas the identity of the receptors for HCV and HCMV is far from clear. Viruses may also use alternative receptors (e.g. CXCR4 for some HIV strains) or different receptors in different cell types (e.g. epithelial GalCer for HIV). Furthermore, additional molecules (coreceptors) may be required for entry (such as chemokine receptors for HIV, CD13 for HCMV and the scavenger receptor B1 for HCV). Finally, as recently shown, the efficiency of viral entry is conditioned by multiple cellular factors that may enhance infection in *cis *or in *trans*.

Bartenschlager *et al*. presented a review of recent findings on HCV replication. Bartosch *et al*. then discussed their observation that human serum components increase the infectivity of HCV pseudoparticles. These components are primate-specific as sera from chimpanzees and rhesus monkeys increase HCV infectivity whereas sera from rabbits and cows do not. The enhancement of infectivity by serum components has been described for other viruses (Ebola virus). However, for HCV, this effect is not mediated by immunoglobulins or complement, but is instead due to interplay between the HVR1 region of the HCV E2 glycoprotein, SR-B1 and serum HDL. Coating HCV with such serum lipoproteins may also help the virus to evade neutralizing antibodies.

Bomsel *et al*. discussed their observation that HIV-1 transcytosis is more efficient with infected cells than with cell-free virus. They showed that the transcytosis induced by HIV-1-infected cells involves the adhesion-mediating RGD-containing protein. They also showed that the scaffolding protein HSPG Agrin is expressed at the apical epithelial surface and acts as an attachment factor for HIV-1, by interacting with the gp41 P1 lectin site, synergizing binding to the epithelial receptor galactosyl ceramide. GalCer is also expressed in immature dendritic cells (iDCs) and may mediate the internalization of HIV and its transfer to CD4+ cells in a lipid raft-dependent manner, independently of DC-SIGN. The role of DC-SIGN in viral retention and enhancement is not fully understood and depends on the cell line studied. The data presented by Nobile *et al*. suggest that HIV-1 X4 viruses can replicate in iDC and Raji-DC-SIGN cells, but only covertly and slowly. They show that transfer from iDC or DC-SIGN+ to CD4+ T cells occurs during the first few hours after exposure to virus, whereas only replicating viruses (i.e. not single-cycle virions) are transmitted several days after exposure, suggesting that long-term transmission is associated with replication in DC rather than with the retention of infectious particles through DC-SIGN.

#### The fusion step of entry

After interaction of the HIV-1 envelope surface glycoprotein with host cell receptors and coreceptors, the fusogenic transmembrane glycoprotein undergoes a series of conformational changes that lead to the insertion of the fusion peptide into the membrane of the target cells, bring the viral and cellular membranes in close proximity, and finally triggering virus-cell membrane fusion. Each of these steps provides an opportunity to prevent viral entry. Several agents targeting viral entry are currently being developed, and one – T-20 or Efarvirtide – has been approved by the US Food and Drug Administration for use as an antiretroviral drug. Tokunaga *et al*. have studied a highly fusogenic proviral HIV1-pL2 clone, with a view to identifying the amino acids responsible for this enhancement of fusion activity. By making recombinant envelopes combining parts of pL2 with parts of the less fusogenic pNL4.3, they identified glycine 36 of gp41 as essential for fusion enhancement. This glycine is conserved in almost all HIV-1 isolates, but not in the prototypic pNL4.3. In pNL4.3, an aspartic acid is found in position 36, preventing the correct formation of the helix hairpin, thereby decreasing fusion activity and infectivity. Interestingly, HIV-1 mutants escaping the effects of the fusion inhibitor T-20 frequently present variations in amino acid 36 of gp41.

Münch *et al*. constructed peptide libraries from the hemofiltrates of patients with chronic renal failure and searched for antiviral peptide agents involved in the innate antiviral response. They identified a 20-residue peptide – VIRIP (virus inhibitory peptide) – that specifically inhibited infection with various HIV-1 isolates. This peptide, corresponding to a C-terminal fragment of 1-antitrypsin (the most abundant circulating serine protease inhibitor), seems to exert its antiviral effects by direct interaction with the gp41 fusion peptide.

Hovanessian *et al*. identified a caveolin-1-binding motif within the ectodomain of gp41. This motif is conserved in all HIV-1 isolates and seems to be functional, as gp41 is found complexed with caveolin in infected cells. These researchers designed peptides (CBD1) corresponding to the consensus domain of gp41 and showed that these peptides bound caveolin-1 specifically. The peptides also elicited the production of specific anti-CBD1 antibodies, which inhibited the infection of primary CD4 cells by laboratory and primary HIV-1 isolates. The antibodies act at two different steps: they prevent the infection of cells by HIV particles and aggregate gp41 at the plasma membrane of HIV-1-infected cells, resulting in the production of defective particles. Unlike other gp41 epitopes, the CBD1 epitope is not a transient conformational epitope.

#### Receptors and signaling

Does the interaction between HIV-1 envelope glycoprotein and cell receptors, including the chemokine receptors CCR5 and CXCR4, for viral entry induce signals relevant to viral replication and cell function? This is a key issue in this field. Chakrabarti *et al*. presented convincing data showing that X4-tropic Env gp120, whether monomeric or in its natural conformation on inactivated HIV-1 virions, triggers a signaling array similar to that induced by SDF-1, the natural ligand for CXCR4, in unstimulated primary CD4 T cells. At concentrations (200 nM) close to the Kd for CXCR4, HIV-1 gp 120 efficiently activates Ga proteins and induces calcium mobilization, and activation of the MAP and PI3 kinase pathways. Inactivated virus and gp120 trigger CXCR4-dependent actin cytoskeleton rearrangements and cell chemotaxis. Thus, gp120 may function as a chemokine, inducing structural and functional changes favoring viral entry and replication, and may affect the trafficking and functional responses of unstimulated CD4+ T cells. The interactions between CD4 and CCR5 at the plasma membrane and their role in HIV-1 entry were explored by F. Bachelerie and coworkers, using FRET on living cells transduced with both receptors. The results of this group suggest that the two molecules are colocalized on the cell membrane, where they interact in a stable fashion. The disruption of this interaction inhibits R5 HIV-1 infection. The same team also presented data on the relationships between CCR5 activation, signaling and β-arrestin-mediated endocytosis and chemotaxis, suggesting that different CCR5 structural determinants may be involved in these responses.

### Session 2: Viral sanctuary

***Chair: ***R. Pomerantz; ***Keynote Lecture: ***"HIV residual disease: The main barrier to viral eradication in the era of HAART" by *R. Pomerantz*.

Roger Pommeranz introduced the session with his keynote speech on "Viral reservoirs as major obstacles for viral eradication despite effective highly active antiretroviral therapy (HAART)". The combination of at least 3 different antiretroviral drugs in the clinical management of HIV-1 infection has improved the prognosis of HIV-1 infected patients. However, despite this therapy, HIV-1 has not been eradicated, at least partly due to latent HIV-1 replication occurring in the resting TCD4+.

#### Combination therapy to induce out of latency

The HIV-1 replication cycle includes a large number of possible stages for latency and persistence. Two mechanisms have been described: pre-and post-integration into the human genome. A large body of data has accumulated to indicate that the cells of HIV-1- infected patients may contain proviral DNA but produce only a small amount of viral RNA. In pre-integration HIV-1 latency, differences in latently infected cells may be observed, depending on the severity of disease. The virus may maintain cellular latency via various molecular mechanisms, which may depend on cell type. Understanding the basis of viral latency would make it easier to design new strategies for viral eradication. Because resting CD4+ T-cells are a major component of the reservoir of circulating cells in vivo, Pomerantz suggested that persistently infected cells should be activated in order to purge the viral reservoirs, making it then possible to control the production of new viruses by HAART. For example, IL-2 treatment could be combined with d4T/3TC/Efavirenz; or treatment with OKT3 anti-T cell receptor monoclonal antibody could be combined with ddI. However, experimental data have shown viral rebound after cell stimulation with IL-2, for example. The new viruses produced upon activation came from follicular dendritic cells, lymph nodes, cells in sanctuary sites and other tissues. Most of the HIV produced from reservoirs and during rebound are defective in the V3 sequence of the HIV-1 envelope gene. Resting cells reflect the state of the immune system. In contrast to the results obtained with IL-2, therapeutic strategies based on the stimulation of cells with IL-7 associated with HAART have yielded promising results. IL7 alone is indeed a more potent activator of latent infected cells than IL-2. Most of the newly produced viruses had a CXCR4 and CCR5 phenotype.

Although these approaches show promise for circulating resting CD4+ T-cells, new strategies must be defined to target novel pharmacological drugs to viral sanctuaries such as the brain or testes. A combination of both approaches may facilitate eradication of the viral reservoir in HIV-1-infected individuals.

Massips et al. have followed three HIV-1-infected patients with viral loads below the detection threshold and who have been on HAART for 7 years. Viral DNA was detected in the memory and naive CD4+ T-cell subsets and in CD14+ monocytes, but not in CD56+CD3-NK cells. Phylogenetic analysis demonstrated that the various types of blood cell in two of the three patients harbored genetically different quasispecies. This suggests that the virus populations within each type of blood cell evolved independently and may originate from difference sources (differences in CXCR4 and/or CCR5). Real cellspecific compartmentalization of residual virus populations is thus observed in patients on HAART. For instance, in one of the three patients investigated, CCR5 variants were found in naive CD4+ T-cells and in memory CD4+ T cells. However, both CXCR4 and CCR5 variants were present in CD14+ monocytes. In another HIV-1-infected patient, CXCR4 variants were found in CD14+ monocytes and in naive CD4+ T cells, whereas both CXCR4 and CCR5 variants were found in resting memory CD4+ T cells.

Ivan Hirsh et al. reported that CCR5 HIV-1 variants predominantly infect CD62Lnegative memory T cells, which selectively express the CCR5 receptor. The predominance of CXCR4 HIV-1 variants in less differentiated memory CD4+ T cells may be related to their activation state, as suggested by the expression of both CD45RA and CD45RO molecules on their membrane. In addition, most viruses isolated from peripheral blood resting cells of HIV-1-infected patients with levels of viral RNA in plasma below the detection threshold have few mutations conferring drug resistance. The CCR5 HIV-1 variants, which predominantly infected memory T cells, were found to be resistant to nucleoside reverse transcriptase inhibitors (NRTIs) such as zidovudine and lamivudine. As pointed out by J. Ghosn and colleagues, resistance mutations acquired by HIV-1 during primary infection may correspond to the dominant viral population, and are archived in cellular reservoirs at an early time point, despite treatment. In summary, virological failure in the resting memory CD4+ T cells, the emergence of a dominant pool of HIV-1-resistant virus very early in primary infection and the difficulties involved in getting drugs into viral sanctuary sites, once again raise questions as to the best combination of approaches for eradicating HIV-1 from infected individuals.

#### HCV and IFN-alpha

Feray et al. reported the effect of interferon-alpha in patients infected with hepatitis C virus (HCV). Differences in the composition of HCV quasispecies between plasma and peripheral blood mononuclear cells (PBMCs) suggest that PBMCs support viral replication. The frequency of compartmentalization in 119 naive patients chronically infected with HCV was determined and found to be correlated to virological response to inteferon-alpha. A significant proportion of HCV patients responding well to IFN-alpha treatment were found to be coinfected with variants not found in plasma. This relationship was independent of route of infection, plasma genotype and duration of infection.

### Session 3: Restriction of viral replication

***Chairs: ***B. Cullen and D. Moradpour; ***Keynote Lecture: ***"Defensive arts: innate intracellular immunity against retroelements" by *D. Trono*.

#### Innate and adaptive immunities to HCV in the host

Most viral infections are successfully controlled by conventional innate and adaptive immune responses developed by the host. Viruses such as HIV, HCMV and HCV are able to persist in their host in the long term thanks to multiple strategies aimed at shutting down antiviral defenses. However, one major difference between HIV and HCMV on the one hand, and HCV on the other, is that HCV infections may, in some cases, resolve spontaneously or under treatment. Critical immunological events may thus take place early in viral infection that lead to viral clearance. Our understanding of these early events in the antiviral immune response has led to great efforts in recent years to diagnose HCV infection during the acute phase. This trend was illustrated by the two presentations on HCV in this session. F. L. Cosset focused on the analysis of neutralizing antibodies in a cohort of 17 individuals acutely infected with a single nosocomial outbreak strain of genotype 1b HCV. Neutralizing activity, evaluated by assessing the ability of the patients' sera to inhibit the infection of HuH7 cells by HCV pseudotyped particles, was monitored, together with viral load and phylogenetic analysis of the predominant viral strains was also carried out. The patients studied could be divided into two subgroups on the basis of the infecting genotype 1b strains. Group 1, infected with strain A, showed a very large decrease in viral load within nine weeks of infection whereas group 2, infected with strain B, maintained high viremia. One major finding of this study was that group 1 patients display potent neutralizing activity that is inversely correlated with viremia. The sera from group 2 patients were found to facilitate infection with HCVpp rather than neutralizing such infections. This study strongly suggests that neutralizing antibodies are involved in the control of HCV infection – an observation in apparent contradiction with recent reports [1-3] and with data reported by C. Bain in this same session. Bain's study was performed on a cohort of seven intravenous drug users acutely infected with genotype 3 HCV treated with pegylated IFN-alpha. In this study, the neutralizing activity of the patients' sera neatly paralleled titers of antibodies specific for the envelope E2 glycoprotein. However, these neutralizing antibodies were found both in patients who responded to antiviral therapy and in those who did not, calling into question the role of neutralizing antibodies in therapeutic resolution of acute HCV infection. Longitudinal analysis of T-cell immune responses did not result in the identification of immune correlates of the therapeutic resolution of acute HCV infection. T-cell responses were surprisingly weak throughout follow-up, as shown by comparison with recently published data [4,5] but were improved by treatment with immunomodulators. The initiation of IL-2 treatment strongly increased not only the vigor, but also the breadth of HCV-specific immune responses, revealing significant reactivity to the newly described alternative reading frame protein (ARFP) of HCV, in particular. However, therapeutic recovery from HCV infection could be achieved in the presence of T-cell suppressive mechanisms, suggesting that the presence of immunosuppressive T cells is not in itself responsible for therapy failure and subsequent chronic infection and that these cells probably modulate detrimental immune responses to maintain persistently low levels of liver inflammation in chronic HCV infection. The existence of immune responses to ARFP provides further evidence that this protein is synthesized in natural HCV infection and, like conventional HCV antigens, is expressed during the early steps of HCV infection. Together with published studies, these two presentations highlighted the difficulties involved in identifying immune correlates of viral clearance in a viral infection that may resolve spontaneously.

#### Intrinsic host immunity to HIV

In contrast to what has been reported for HCV, some HIV-infected individuals may control disease progression, but they never eliminate viral infection altogether, suggesting that the conventional immune system is unable to control viral replication. In addition to conventional innate and acquired immune responses, complex organisms have developed so-called "intrinsic" immunity, mediated by constitutively expressed restriction factors that efficiently prevent or limit viral infections [6]. Two major classes of factor have been shown to restrict retroviral infections by blocking incoming retroviral particles (Fv1 and TRIM5a) or by the specific deamination of dC residues to generate dU, leading to the hypermutation of viral DNA and blocking viral replication (APOBEC3; class: cytidine deaminases). Obviously, viruses have evolved strategies to overcome these restriction mechanisms. D. Trono, in the opening lecture of the session, summarized recent data on the cytidine deaminase superfamily, mostly focusing on APOBEC3G [7]. This restriction factor, primarily found in T lymphocytes and macrophages, is packaged into HIV virions in the absence of Vif protein, via specific interaction with the NC/p6 domain of the Gag polyprotein precursor (B. Cullen) or non-specific RNA binding [8]. Upon the infection of new target cells, APOBEC3G deaminates the nascent minus strand DNA, resulting in a less stable uracyl-containing minus-strand DNA, which is degraded or yields hypermutated plus-strand DNA liable to encode defective viral proteins. In the presence of Vif, APOBEC3G is targeted for proteasome degradation. Bet protein, derived from primate foamy virus, can partially rescue Vif-deleted virions (B. Cullen). APOBEC3G has been shown to block a wide range of retroviruses and unrelated viruses such as hepatitis B virus (HBV) [9]. If hepatoma HuH7 cells are cotransfected with a plasmid containing the HBV genome and a plasmid encoding APOBEC3G, intracellular levels of core-associated HBV DNA are significantly lower than those in cells transformed with the viral genome alone. Although this effect is inhibited by HIV-1 Vif, catalytically inactive APOBEC3G continues to have an inhibitory effect on HBV DNA, suggesting that APOBEC3G may act on HBV and retroviruses via different mechanisms. However, one unresolved question concerns the potential relevance of such an interaction as APOBEC3G is expressed in lymphoid cells and HBV mostly infects hepatocytes. Anti-HIV-1 treatments targeting Vif protein may eventually come out of this work.

#### RNAi targeted to HIV

R. Benarous presented work on another antiretroviral strategy, the use of RNA interference to block the interaction between HIV integrase and a cellular protein, the lens epithelium-derived growth factor/transcription coactivator p75 (LEDGF/p75) protein. HIV-1 replication is strongly inhibited by the presence of siRNA targeting the 3' end of the LEDGF coding region, suggesting that this protein is required for HIV infection. Further experiments with HIV integrase (Gln168) mutants displaying defective HIV-1 DNA integration, demonstrated the involvement of LEDGF in the targeting of integrase to chromosomes. HIV-1 can infect the central nervous system (CNS), where it causes progressive cognitive and motor dysfunctions. Astrocytes have been shown to be target cells for HIV-1 in the CNS but these cells allow only limited replication of HIV-1. They can also be infected with HIV-1 *in vitro *but such infections are generally of very low and transient productivity, suggesting that astrocytes may contain a factor that restricts HIV-1 replication.

#### Rev and RNA transport

In this session, S. Kramer-Hämmerle reported an abnormal distribution of HIV-1 Rev in astrocytes, with a blockade of its nucleocytoplasmic shuttling function leading to the inhibition of nuclear export of HIV-1 mRNAs. Using a cDNA library from astrocytes, a double-hybrid strategy in yeast and then in mammalian cells, Kramer-Hämmerle identified a cellular factor – 16.4.1 – that colocalized with Rev in transfected cells. This factor also interacted with an exportin, CRM1, a member of the karyopherin family of nucleocytoplasmic transport factors and a cellular cofactor for the Rev-dependent export of HIV-1 RNAs. 16.4.1, which is probably part of a larger protein, reduces Rev activity. These data illustrate the huge diversity and complexity of mechanisms developed by these two viruses for the establishment of chronic infection. However, these two viral infections differ primarily in that HCV-infected patients, unlike HIV-infected patients, may recover spontaneously from infection. This may explain why the study of conventional immune responses has always been a major research field for HCV whereas HIV research is gradually turning to the investigation of more intrinsic interactions between host and viral proteins.

### Session 4: Viral infection and innate immunity

***Chairs: ***C. Soderberg-Naucler & L. Zitvogel; ***Keynote Lecture: ***"Immunopathology of prion infection" by *A. Aguzzi*

#### Prions

This session began with a keynote lecture by A. Aguzzi, presenting data on two aspects of prion infection. He first presented an immunointervention strategy for modulating the course of scrapie in mice, based on a chimeric PrP molecule consisting of two PrP fused to the constant fragment of an IgG (PrP-Fc2). The aim was to interfere with the PrPsc – PrPc interaction, which results in there being two PrPsc conformers and spreads "infection", as a means of limiting disease. Aguzzi's team hypothesized that an Fc-linked dimer of PrPc would interact with PrPsc without transconformation, thereby blocking prion progression. Such an interaction was demonstrated to exist as PrP-Fc2 precipitated PrPsc from diseased brains. Crossing WT mice and transgenic mice expressing PrP-Fc2 delayed the onset of scrapie (by up to 150 days) and decreased PrPsc accumulation. Moreover, PrP-Fc2, which normally settles in the bottom layer of membrane fraction gradients, was redistributed to the raft layer, which is the site of PrPsc is in infected brain preparations. Nevertheless, the delay in scrapie onset may not be entirely due to higher levels of PrPsc clearance through the reticulo-endothelial system, as the Fc fragment was deleted from its FcgR interaction site. These encouraging data led to the transfer of PrP-Fc2 into WT mice brain via lentiviral vector, which conferred clinical resistance to scrapie for up to 265 days. The PrP-Fc2 transferred by the lentivirus decreased astrogliosis in the injected hemisphere whereas the contralateral hemisphere continued to displaye strong GFAP reactivity. The PrPsc signal was cleared only near the injection site. The protection conferred by PrP-Fc2 requires central expression, as peripheral injection is not protective, although PrP-Fc2 expression can be targeted to oligodendrocytes, a cell type not infected by prions, in the periphery.

Aguzzi then rapidly presented data for transgenic mice displaying targeted tissue-specific expression of lymphotoxin antibody. In these mice, which displayed tertiary lymphoid tissue development in the liver, the kidney or the pancreas, the replication responsible for infectivity occurs in these organs. This raises questions of food safety, if animals with inflammation sites are used for meat, but may also open up new possibilities for the use of peripheral preventive strategies such as PrP-Fc2 injection during the invasion phase of spongiform encephalopathies.

#### NK cells and HCV, HIV, and HCMV

The session then moved on to more conventional viruses and dealt with the effects of HCV, HIV and HCMV actions on natural killer cells and monocytes/macrophages. U. C. Meier presented comparative data on NK cell modulation in response to HCV and HIV infection. The major subpopulation of NK cells in uninfected humans is CD3-/CD56 dim NK cells. These cells are highly cytolytic and display strong NK receptor expression, and low levels of trafficking and cytokine production. The minor CD3-/CD56 bright subpopulation displays the opposite phenotype with respect to these characteristics. In response to HCV and HIV infections, the number of NK cells in the blood decreases and there is a shift toward the CD56 bright subpopulation, with no change in CD57 expression on NK cells. This results in a decrease in the percentage of perforin-bright NK cells in favor of perforin-dim cells. In HCV patients, this decrease was shown not to correspond to NK cell accumulation in the liver. The response of NK cells to HCV and HIV infections differed in that interferon production under IL12 + IL18 stimulation decreased in the NK cells of HIV patients but increased in those of HCV patients. The decrease in frequency of NK cells may be the consequence of a loss of IL15 expression, as the serum concentration of this cytokine is low in both infections, or of an impaired response to the IL15 survival signal. Such an impaired response to IL15 was demonstrated only in HIV infection, in terms of survival and cytolysis.

#### HCV, HCMV and monocyte activation

Assessment of the effect of HCV and HCMV on monocyte activation and differentiation as a means of estimating viral persistence was the subject of two talks, by P. Balard and S. Gredmark. HCV persistence is thought to be associated with a Th2 bias, which is demonstrated by a decrease in IL-12 production and an increase in CD36 membrane expression on monocytes. Chêne *et al*. showed that HCV core protein induces the overproduction of PGJ2 by the PLA2 – Cox2 cascade, with Cox2 overproduced. PGJ2 is a ligand for PPARl, which is activated in HCV-core-treated monocytes, and involved in CD36 and IL-12 modulation. These results are consistent with the notion that the HCV present in the patient's serum may establish a chronic infection by inducing an M2 orientation of monocyte activation, leading to a biased T-cell response. Monocytes-macrophages are also critical to HCMV infection, as this virus can be reactivated *in vitro *from macrophages. HCMV strategies for escaping immune surveillance include decreases in the expression of MHC class I and class II molecules, the impairment of T-cell activation, and a decrease in NK cell-mediated lysis. Gredmark *et al*. found that a suspension of HCMV inhibited the differentiation of monocytes into mature macrophages, resulting in the production of monocytoid cells with impaired migration and phagocytosis and low levels of β-chemokine production. This inhibition was achieved with inactivated HCMV, but not with HCMV suspension supernatant; nor was it reproduced with HIV or measles virus. The viral effector was identified as the gpB protein of HCMV, which binds to CD13 and signals by means of Ca^2+ ^flux, through this receptor. CD13 is an N-aminopeptidase involved in monocyte-macrophage adhesion and migration. Using monoclonal CD13 antibodies, Gredmark were able to mimic or to anatagonize the effect of HCMV on macrophage differentiation, depending on the clone used.

These two talks strongly suggested that monocytes-macrophages are, together with NK cells, a major target for the prevention of viral persistence and infection chronicity. However, viruses may use several different strategies, involving numerous mechanisms to establish chronic infections.

### Session 5: Chemokines and inflammatory cytokines

***Chairs: ***K. Klenerman and G. Poli; ***Keynote Lecture: ***"CD4 T-cell homeostasis in HIV infection: role of the thymus" by R. Sekaly.

#### CD4 T-cell homeostasis in HIV infection: role of the thymus

In chronic viral infections, CD4+ T-cell responses are associated with disease control. R. Sekaly reported stronger proliferative HIV-specific CD4+ T-cell responses in aviremic than in viremic patients. Long-term CD4+ T-cell memory depended on IL-2-producing CD4+ T cells whereas cells producing only IFN-γ were short-lived. Sekalt characterized the *ex-vivo *phenotype of CD4+ T cells in more detail by genomic and proteomic analysis, and identified genes differentially expressed along the CD4+ T-cell differentiation pathway: 1) TOSO, which inhibits Fas- and TNF-mediated apoptosis, and PIM2 and DAD1 were more strongly expressed in naive and central memory CD4+ T cells than in effector/memory and effector CD4+ T cells. These genes were also expressed more strongly in samples from healthy donors than in samples from viremic patients; 2) Conversely, Rab27a, which indicates the activation state of T-cell maturation, was expressed more strongly in effector and effector/memory CD4+ T cells than in naive and central memory CD4+ T cells. These data provide new insights into CD4+ T-cell homeostasis during HIV infection.

#### Cytokine production in the livers of HCV+HIV- and HCV+HIV+ individuals

As cytokines play a crucial role in controlling the immune responses against viral persistence, G. Paranhos-Baccala *et al*. measured intrahepatic levels of IFN-gamma, TNF-alpha, TGF-β, IL-2, IL-4, IL-8, IL-10 and IL-12p40 by real-time PCR in 12 HCV+HIV- and 14 HCV+HIV+ individuals. They showed that the detection rates for individual cytokines were higher for the HCV+HIV- group than for the HCV+HIV+ grou. However, only the detection rates for TNF-alpha, IL-8 and IL-10 differed significantly between the two groups. Moreover, median levels of IFN-gamma, IL-8 and IL-10 were significantly higher in the HCV+HIV+ group. This study demonstrated the existence of a global defect in cytokine signaling in HCV+HIV+ individuals, which may contribute to HCV persistence.

#### HIV interactions with other pathogens in coinfected human lymphoid tissues

Recent epidemiological studies have reported examples of of the inhibition of HIV replication by microbial interactions. In a study of *ex vivo *-infected human lymphoid tissue, L. Marogolis *et al*. showed that two microbes (measles virus (MV) and *Toxoplasma gondii *(TG)) inhibited the replication of both CXCR4-tropic (X4) and CCR5-tropic (R5) HIV-1. This inhibitory effect was particularly marked for R5 virus and was mediated by a parasite-encoded cyclophilin, C18, in TG-infected tissues, and by a CC chemokine, RANTES, in MV-infected tissues. These microbes were also found to display a moderate cytopathic effect on lymphocytes, decreasing the number of R5 and X4 HIV-1 targets in co-infected tissue. This study highlighted the crucial role of the cytokine/chemokine network in interactions between microbes in the human host.

#### Early induction of an anti-inflammatory environment may temper T-cell activation during SIVagm infection

During primary SIVagm infection, African green monkeys (AGM) can display a transient decline in CD4+ T-cell counts together with transient T-cell activation until the end of primary infection. Cytokine gene expression was assessed in a longitudinal studycarried out by Ploquin et al., before infection and at intervals of two to three days during primary infection (PI), and then regularly until day 430 postinfection. The following observations were made in SIVagm-infected AGMs: 1)A significant increase in TGF-b1 and Foxp3 gene expression beginning in the first week after infection, coinciding with expansions of the populations of CD4+CD25+ and CD8+CD25+ T cells; 2) An increase in IL-10 gene expression during the 2nd and 3rd week p.i, with no change in TNF-alpha gene expression at any point in the study; 3) Changes in the plasma concentration of cytokines correlated with gene expression changes. In conclusion, the harmful generalized immune activation levels observed during the post-acute phase of SIVagm infections may be controlled by the early induction of anti-inflammatory cytokines, as observed in this study.

#### HIV infection: role of IL-7 in immune reconstitution after HAART or HAART plus IL-2 and preclinical assessment of its therapeutic potential

As plasma IL-7 levels are negatively correlated with CD4 counts during HIV disease progression and antiretroviral therapy, J. Theze suggested that IL-7 is part of a feedback loop regulating the size of the CD4 pool. In this study, plasma IL-7 levels at the start of HAART were found to be positively correlated with an increase in CD4 counts during the first two years of HAART. Plasma IL-7 concentrations increased in HIV-infected patients receiving HAART plus IL-2. Theze assessed the therapeutic potential of IL-7 by studying IL-7R expression in CD4 and CD8 T lymphocytes from three groups of patients (group 1: naive for antiretroviral therapy (plasma viral load > 10,000 copies /ml and CD4 count > 350 cells /mm^3^); group 2 : HAART-treated patients with CD4 > 400 cells/mm^3 ^and plasma viral load < 50 copies /ml; group 3: HAART-treated patients with CD4 counts remaining low (CD4 < 250 cells /mm^3^) despite good control of plasma viral load (< 50 copies /ml)). The major findings of this study were: 1) CD127 was less strongly expressed on CD4 lymphocytes from group 1 and group 3 patients than on those from group 2 patients; 2) CD8+ lymphocytes from HIV-infected patients were mostly CD27-CD45RO+ and CD27-CD45RO-; 3) High viremia was correlated with IL-7R dysfunction, whereas HAART-treated patients recovered a functional IL-7R. These concluded that the IL-7/IL-7R system plays a role in HIV disease and that IL-7 could be used in immune interventions to treat HIV infection.

#### The HIV-1 mediated induction of ET-1 in the CNS increases the secretion of markers of blood-brain barrier failure, which are altered by HIV-1 protease inhibitors, nelfinavir

N. Didier *et al*. suggested that endothelin-1 (ET-1) is involved in the neuropathogenesis of HIV-1 infection because ET-1 levels have recently been shown to be correlated with the degree of encephalopathy in HIV-1-infected individuals. Using a model of the blood-brain barrier (BBB), N. Didier *et al*. showed that the production of ET-1 by brain endothelial cells in response to HIV-1 leads to disruption of the BBB by the pro-inflammatory cytokines (IL-1, IL-6 and IL-8) produced by astrocytes. As proteases play an important role in inflammatory processes, nelfinavir decreases the level of cytokine secretion, and may therefore be useful in HAD.

### Session 6: Dendritic cells and activation of T-cell antiviral responses

***Chairs: ***B. Autran & A. Hosmalin; ***Keynote Lecture: ***"Combat between cytomegalovirus and dendritic cells in T-cell response" by *C. Davrinche*;

#### Combat between cytomegalovirus and dendritic cells in the T-cell response

During HCMV infection, innate (apoptosis, IFNα/β, complement, NK cells and dendritic cells) and adaptive (CD4+, CD8+ and antibodies) immune responses are generated. The main target proteins for CD4 and CD8 T cells are IE1 and pp65 (early proteins). In a model consisting of dendritic cells (DC) cocultured with HCMV-infected fibroblasts, C. Davrinche showed that the fibroblasts rapidly became apoptotic. The DC acquired pp65 from infected fibroblasts via a mechanism requiring cell-to-cell contact and, after 6 hours, DC produced TNFα and IL6. In the presence of PBMC, a large number of pp65-specific CD8 T cells were generated and a peak of IFNγ production was observed 24 h after incubation. DC maturation (upregulation of CD83) was induced by incubation with HCMV-infected fibroblasts, and a peak in CD83 expression was observed after 6 h, with levels decreasing after 48 h and 72 h. This maturation seems to be a prerequisite for efficient T-cell stimulation. C. Davrinche has identified a soluble factor (TGF-β) secreted at a late stage of HCMV infection in fibroblasts that downregulates CD83. He has also shown that the IL10 homolog carried by HCMV interferes with DC maturation and cross-presentation. Overall, the results presented suggested that cross-presentation must occur soon after infection by HCMV to prevent the soluble factor-mediated viral escape mechanism. This may explain why the main target proteins for T-cell responses are IE1 and pp65, which are available early in infection.

#### HIV-1-induced dysfunction of naive CD8+ T cells

D. Favre showed that in the SCID-hu thymus/liver mouse model, HIV infection of the thymus resulted in a CD8 functional defect due to impaired signaling via the TCR complex, with effects on calcium flux and IL-2 responses (cytokine production and proliferation). After the transplantation of a human thymus/liver graft in SCID mice, thymocytes from SCID-hu mice were infected in week 18 with HIV-1 NL4-3, BaL, or primary stocks and the infected animals were compared with mock-infected animals. HIV infection of the thymus induced the upregulation of MHC-I in thymocytes, correlated with increases in HIV RNA levels and the development of single-positive CD8^low ^(SP8) thymocytes. Following polyclonal stimulation (anti-CD3/CD8) *via *the TCR, a significantly weaker calcium flux response and lower proliferative capacity, as measured by CFSE, were observed in SCID-hu thymus/liver mice than in control mice. Thus, in the SCID-hu thymus/liver mouse model, HIV infection results in the selection of CD8^low ^T cells with defective calcium flux signaling. Favre also presented data concerning the activation status of circulating CD8^+ ^T cells from 40 HIV-1-infected patients at various stages of the disease. In patients with progressive disease, a decrease in CD8^+ ^naive (CD45RA^+^CD27^+^) T-cell counts was observed, with low levels of CD8 expression, associated with chronic immune activation, as assessed with the CD38 marker. A dysfunction in calcium flux and IL-2 responses is also observed in patients with progressive HIV disease. In conclusion, the CD8^low ^T cells observed after experimental HIV infection of the thymus and in the peripheral blood of patients with progressive HIV disease seem to display MHC-I upregulation and defect in signaling across the TCR, associated with chronic immune activation (CD38). Fabre suggested that the higher density of MHC-I on cells in the thymus might lead to high-avidity interactions with TCRs on developing thymocytes and hence to supranormal levels of negative selection, but it remains unclear how these CD8^low ^T cells are generated. Such dysfunctional CD8^low ^T cells would contribute to the profound immunodeficiency associated with HIV disease progression.

#### Role of HIV-1 Nef in viral replication in lymphocytes

The results presented by Nathalie Sol-Foulon demonstrated a requirement for ZAP70 for efficient HIV replication in Jurkat cells and the severe impairment of replication in Nef-deleted virus in Zap-deleted Jurkat cells. In these experiments, Jurkat cells or PBLs were infected with a wild-type HIV or Nef-deleted HIV and stimulated by PMA iono or superantigen. IL-2 production was then evaluated. Sol-Foulon showed that HIV infection increased activation (as assessed by determining IL-2 production) in response to T-cell stimulation *via *the TCR or the MAP kinase signaling pathways. Infection with wild-type HIV or Nef-deleted HIV had no significant effect on IL-2 production (53% and 43%, respectively) so Nef does not significantly affect this process. The absence of ZAP70 is known to cause a major defect in the TCR. HIV replication is strongly affected in Zap-deleted Jurkat cells but it is unclear which step of the viral cycle is affected and the effects of Zap on viral replication in primary T cells and the links between transduction pathways and HIV replication are unknown: Sol-Foulon is currently investigating these aspects.

#### The extent of CD4+ T cell apoptosis during primary SIV infection is predictive of the rate of progression to AIDS

J. Estaquier showed that the rate of CD4+ T-cell apoptosis was correlated with subsequent viremia levels whereas levels of CD8+ T cells were not. In rhesus macaques experimentally infected with the pathogenic SIVmac251 isolate, peak numbers of apoptotic cells in the lymph node T-cell areas were significantly higher in future rapid progressors than in the slow progressors during the first two weeks of infection. No correlation was found between the rate of viral replication within lymph nodes and the extent of FasL-mediated apoptosis in CD4+ T cells. The mechanism of apoptosis seems to be independent of the caspase and AIF pathways. The role played by mitochondria was also evaluated in SIVmac251-infected macaques and the results presented indicated that the Bak gene is involved in SIV-mediated CD4+ T cell apoptosis. Estaquier concluded that memory T cells are lost early in infection and that levels of apoptotic CD4+ T cells are predictive of disease progression.

#### A T-cell based HCV vaccine capable of blunting acute viremia and protecting against acute and chronic disease induced by heterologous viral challenge in chimpanzees

Alfredo Nicosia presented his results for HCV-vaccination with an MRK adenovirus at weeks 0 and 25 and a DNA EP boost in week 35. Chimpanzees were challenged with a heterologous virus in week 49, and the vaccination was shown to have elicited potent, broad-range and durable effector T-cell responses. The immunogen used was from a non structural region of HCV corresponding to genotype 1b, the most frequent strain in USA and Europe. The challenge involved H77, corresponding to a genotype 1a. In this study, five animals were vaccinated and five others received the control vector. Specific IFNγ-CD8+ responses were maximal in week 37, after the booster. Polyspecific HCV- CD8+ responses were detected in peripheral blood and in the liver. These specific immune responses, induced by vaccination, were also elicited by the with challenge strain, demonstrating cross-reaction. Nicosia showed that eight weeks after challenge, viral load in vaccinated animals was less than one hundredth that in control animals (*P *= 0.009). He also demonstrated an absence of liver damage in vaccinated animals, whereas ALT and GGT levels were high in control animals. He concluded that this vaccine can prevent hepatitis and protect animals against chronic infections caused by heterologous viruses.

#### Cross-presentation by dendritic cells of HIV antigens from live infected CD4+ T lymphocytes

e Hosmalin showed that dendritic cells (DC) can capture, and cross-present to specific-CD8+ T cell lines, HIV antigens from live, infected cells as efficiently as antigens from apoptotic infected CD4+ T cells. When MDDC + LPS were cultured with various sources of HIV antigens (peptides from Gag, RT, free virus, CD4+ T cell lines infected with HIV) and presented to CD8+ T cell lines specific for Pol 476–484, the cross-presentation of HIV antigens from apoptotic infected CD4+ T cells was more efficient than direct DC infection or other sources of HIV antigens. Hosmalin also presented other data, showing that similar levels of cross-presentation are also observed in live infected CD4+ T cells. She performed similar experiments with live infected CD4+ T cells and *ex vivo *PBMC from HIV-infected patients. In HIV-infected patients, circulating CD8+ T cells recognized cross-presented HIV antigens from live infected T cells. Thus, anti-HIV immunity begins before the induction of apoptosis. Moreover, the proportion of CD83+ mature DC increased when DC were incubated with primary CD4+ T cell blasts, whether apoptotic or not, and independent of HIV infection. Hosmalin concluded that, during HIV infection, live or apoptotic HIV-infected T lymphocytes can supply antigens and costimulation signals for MHC class-I-restricted presentation by DC or induce tolerance in patients with low CD4 counts and impaired CD4 T-cell functions.
